# 2′-3′-Cyclic Nucleotide 3′-Phosphodiesterase Inhibition by Organometallic Vanadium Complexes: A Potential New Paradigm for Studying CNS Degeneration

**DOI:** 10.3390/brainsci11050588

**Published:** 2021-04-30

**Authors:** David C. Platt, Jonathan Rink, Kamaljit Braich, Craig C. McLauchlan, Marjorie A. Jones

**Affiliations:** Department of Chemistry, Illinois State University, Normal, IL 61790-4160, USA; dcplatt@ilstu.edu (D.C.P.); jonathanrink@gmail.com (J.R.); kamalbraich25@gmail.com (K.B.); ccmclau@ilstu.edu (C.C.M.)

**Keywords:** 2′-3′-cyclic nucleotide 3′-phosphodiesterase, enzyme inhibition, myelin, vanadium complexes, myelin marker enzyme

## Abstract

The enzyme, 2′-3′-cyclic nucleotide 3′-phosphodiesterase (CNPase) has been known for over fifty years. Nevertheless, the roles this membrane-bound enzyme play have yet to be described completely. Recently, there has been renewed interest in the study of this enzyme due to studies that suggest that CNPase plays a role in the mediation of cellular inflammatory responses in renal and nervous system tissues. Also, this enzyme, found in oligodendrocytes of the nervous system, has been reported to participate in significant regulatory changes associated with age which may be involved in age-related CNS degeneration. Consequently, development of CNPase inhibitors is of interest and should aid in the study of this, as yet, poorly understood enzyme. In this work we utilized a spectrophotometric enzyme assay to determine the effect a panel of organo-vanadium complexes had on isolated hamster myelin CNPase activity. Our group has now identified several potent in vitro CNPase inhibitors that could prove useful in clarifying the important roles of this enzyme.

## 1. Introduction

Since its discovery in rabbit central nervous system tissue in the 1960s, the physiological role of 2′,3′-cyclic nucleotide phosphohydrolase (EC # 3.1.4.37; CNPase; phosphodiesterase) has been uncertain [1]. This enzyme family is considered to be myelin marker enzymes [2,3] and one of the major enzymes in myelin [4]. However, this enzyme activity has also been reported in other membranes, such as those from erythrocytes [5] and rat liver and heart mitochondria [6]. Jackson et al. [7] have also reported that this enzyme is found in kidney and speculated that it has a role in acute kidney injury. Experiments seeking to expand our incomplete understanding of the CNPase enzyme’s role have also yielded some exciting results surrounding its function in certain neuropathies. Reports of CNPase involvement with the pathological demyelination of axon terminals seen in multiple sclerosis (MS) patients suggest this enzyme plays a role in the progression of this and other neurological disorders exhibiting demyelination [8]. Erythrocytes from multiple sclerosis patients exhibited significantly lower CNPase activity than control erythrocytes [9]. Also, Baburina et al. [4] reported that this enzyme, found in oligodendrocytes of the nervous system, participates in significant regulatory changes associated with age which may be partially involved in age-related CNS degeneration. In another study [10], CNPase activity was found to be increased in activated microglial cells used as a model for brain injury. CNPase ‘knockdown’ experiments in these cells were followed by increases of the inflammatory mediators IL-1β and TNF-α, as well as various reactive oxygen and reactive nitrogen species. These results suggest an anti-inflammatory role of CNPase in injured central nervous system cells [10]. These reports evidence the important role this enzyme plays in various neuropathies and suggest that inhibition of the enzyme may serve a therapeutic benefit. 

Previous studies have reported inhibitors for this CNPase enzyme include bivalent metal ions such as Cu^2+^ and Zn^2+^, mercury salts, methyl xanthine, synthetic polyribonucleotides, 3′ AMP, and heparin, as reviewed by Baburina et al. [4]. Despite a rich history as inhibitors of enzymes including acid, neutral, and alkaline phosphatases as well as phosphoglycerate mutase B, that remove phosphate groups from their specific substrates [11,12,13], vanadium-containing compounds have, as yet, been only modestly studied as inhibitors of CNPase. Mustapha et al. [14] did report that neonatal mice pups exposed to sodium metavanadate via lactation all had lower body weights and reduced locomotor activity and that oligodendrocytes in culture had reduced expression of CNPase when vanadium was in the culture medium. Here, we report the investigation of the inhibitory effects of a series of known vanadium complexes on the CNPase enzyme. 

## 2. Materials and Methods

**Figure 1:**
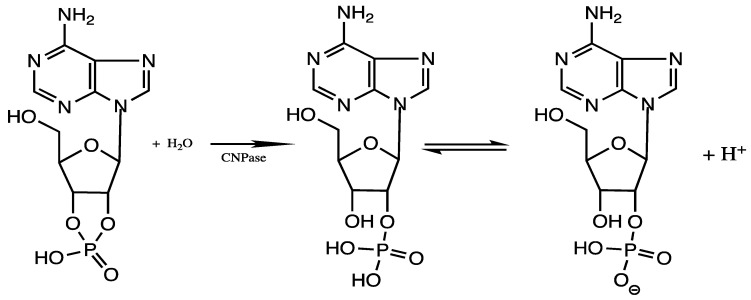
Reaction catalyzed by 2′,3′-CNPase.

To isolate the CNPase enzyme, spinal cord tissue was dissected from hamster spinal columns. Hamster tissues were obtained following euthanasia following the Illinois State University Institutional Review Board (IACUC) protocol #09-2006, 2018 (tissues were a generous gift of Dr. Paul Garris, Department of Biological Sciences, ISU) then frozen and stored at −80 °C until used. This tissue was homogenized in 0.32 M sucrose, and the myelin was extracted via the method of Norton and Poduslo [15] yielding a suspension of hamster myelin. Triton X-100 (Sigma-Aldrich, St. Louis, MO, USA) was added to the myelin suspension to disrupt the cellular membranes and liberate the membrane-bound CNPase enzyme as reported by Lees et al. [16] and Jones and Keenan [17]. Enzymatic activity of this suspension was subsequently assessed by a sensitive colorimetric assay method reported by Jones and Keenan [17]. Bromocresol purple pH indicator was obtained from Sigma-Aldrich (St. Louis, MO, USA) and dissolved in NanoPure water. Sodium orthovanadate (99%) was obtained from Acros Organics (Fair Lawn, NJ, USA) and used without further purification. This was used as a non-organometallic vanadium control. Aliquots of CNPase suspension and pH indicator were adjusted to pH 6.9 with 50 nmoles of NaOH prior to introduction of the substrate, 2′-3′-cyclic adenosine monophosphate (2′,3′-cAMP) (Sigma-Aldrich, St. Louis, MO, USA). Upon hydrolysis of the substrate, H^+^ is produced, according to the reaction scheme depicted in Figure 1, which changes the color of the pH indicator from blue to yellow [17]. This reaction was monitored at 590 nm using a Hewlett Packard 8453 UV/Vis spectrophotometer in the kinetic mode. Thus, the rate of decrease in absorbance at 590 nm with time indicates the rate of reaction in the presence or absence of pre-incubation with vanadium CNPase inhibitor candidates. If other enzymes in the myelin preparation are inhibited by the vanadium complexes prior to addition of substrate, this should have no effect on the rate of CNPase activity. Heat treated myelin (boiled 2 min prior to assay), to denature the 2′,3′-CNPase activity, resulted in no change in slope upon the addition of substrate, indicating that the detected change in absorbance is likely due to the catalytic activity of this enzyme. 

**Figure 2:**
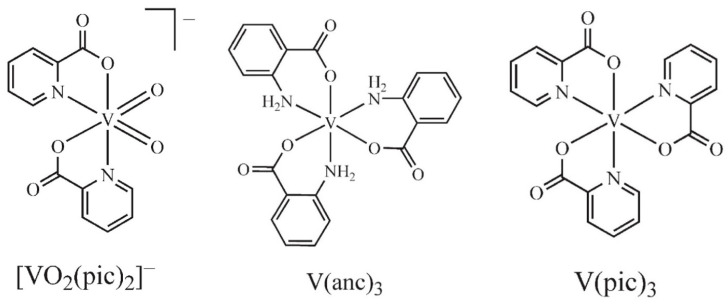
Structures of vanadium complexes found to substantially inhibit CNPase activity in this study.

Organo-vanadium complexes were synthesized as previously reported [12,18,19,20,21] and were tested at 100 µM final concentration, a concentration we have used with other complexes and studies [12,18]. The complexes were pre-incubated with the enzyme for 60 s prior to substrate addition. Results are reported as % enzyme activity remaining relative to no complex addition (set at 100% activity). For the three complexes deemed as good inhibitors (Figure 2), the IC_50_ (µM) values were determined using values from 10 to 100 µM of selected test compound.

## 3. Results and Conclusions

We and others have previously examined the series of vanadium complexes shown in Table 1 as possible enzyme inhibitors with therapeutic potential for several systems [11,12,13,18,19,20,21]. Here, all vanadium complexes tested showed some degree of inhibitory capacity toward CNPase; however, our investigations revealed three potent CNPase inhibitors: V(pic)_3_, V(anc)_3_, and NH_4_VO_2_(pic)_2_. These complexes exhibited IC_50_ values of 20 µM, 30 µM, and 40 µM, respectively. The tabulated results for all the compounds tested are reported in Table 1 and names and full structures are included in the Supporting Information (S1). Interestingly, the most potent inhibitors were not dependent on the oxidation state of the complex added, and were vanadium (III) and (V) species, not the commonly employed vanadium (IV) species such as that found in BMOV and BEOV [20,21]. The ligand system employed also did not seem to play a role, as both picolinate and anthranilate complexes proved to be effective inhibitors. Thus, a combination of steric and electronic effects is likely involved in driving the inhibitor effect. Use of these most effective vanadium complexes can now be applied to other in vitro systems to evaluate roles of this enzyme in various physiological responses. 

## Figures and Tables

**Table 1:** Results from CNPase enzyme assay showing percent of activity remaining in the presence of vanadium complexes (10–100 µM). The complexes demonstrating the greatest degree of inhibition are shown in bold with indicated IC_50_ values. The plots for these three complexes are shown in the Supplemental Materials Figure S2.

Vanadium Complex Tested		% Activity Remaining	IC_50_ (µM)	Synthesis Reference
No complex control		100%	--	--
PO_4_OEt	1	87%	--	19
PO_4_OME	2	48%	--	19
BEOV, VO (ema)_2_	3	73%	--	20
BMOV, VO (ma)_2_	4	82%	--	21
**V (pic)_3_**	**5**	**9%**	**20**	**18**
VO (pic)_2_	6	57%	--	18
**NH_4_** **VO_2_** **(pic)_2_**	**7**	**14%**	**40**	**18**
V (imc)_3_	8	30%	--	18
VO (imc)_2_	9	39%	--	18
NH_4_VO_2_ (imc)_2_	10	52%	--	18
**V (anc)_3_**	**11**	**6%**	**30**	**18**
VO (anc)_2_	12	65%	--	18
NH_4_VO_2_ (anc)_2_	13	82%	--	18
Na_3_VO_4_	14	77%	--	--
(NH_4_)_6_V_10_O_28_	15	36%	--	12

Anc = anthranilate, ema = ethylmaltolate, imc = imidzolylcarboxylate, ma = maltolate, pic = picolinate.

## Data Availability

Data are available through correspondence to M.A.J.

## Supplementary Materials

The following are available online at https://www.mdpi.com/article/10.3390/brainsci11050588/s1, Figure S1: Graphical representation of complexes in Table 1. Figure S2. Plots for estimation of IC_50_ Values.
