# Supplementary material for: 2′-3′-Cyclic Nucleotide 3′-Phosphodiesterase Inhibition by Organometallic Vanadium Complexes: A Potential New Paradigm for Studying CNS Degeneration
Source: Brain Sci. 2021 Apr 30;11(5):588. doi: 10.3390/brainsci11050588 (PMC8147186; doi:10.3390/brainsci11050588)
Supplement: Supplementary file 1 [file brainsci-11-00588-s001.zip › brainsci-1180761-supplementary.pdf]

Supplementary Materials

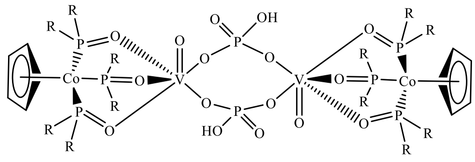

1 R= OEt

bis(μ_2-O,O-phenylphosphato)-hexakis(μ_2-diethylphosphito)-bis(η^5-cyclopentadienyl)-dioxo-di-cobalt-di-vanadium(IV)

2 R= OMe

bis(μ_2-O,O-phenylphosphato)-hexakis(μ_2-dimethylphosphito)-bis(η^5-cyclopentadienyl)-dioxo-di-cobalt-di-vanadium(IV)

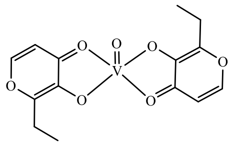

$VO(ema)_2$

diethylmaltolatooxovanadium(IV)

3

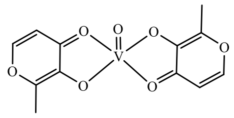

$VO(ma)_2$

dimaltolatooxovanadium(IV)

4

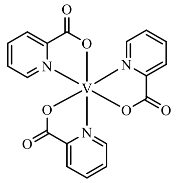

$V(pic)_3$

tripicolinatovanadium(III)

5

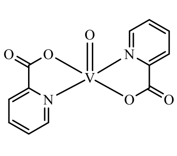

$VO(pic)_2$

oxodipicolinatovanadium(IV)

6

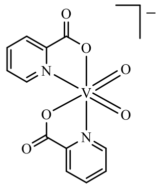

$[VO_2(pic)_2]^-$

dioxodipicolinatovanadate(V)

7

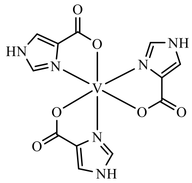

$V(imc)_3$

tri(1H-imidazole-4-carboxylato)vanadium(III)

8

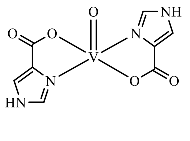

$VO(imc)_2$

di(1H-imidazole-4-carboxylato)oxovanadium(IV)

9

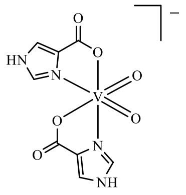

$[VO_2(imc)_2]^-$

di(1H-imidazole-4-carboxylato)dioxovanadate(V)

10

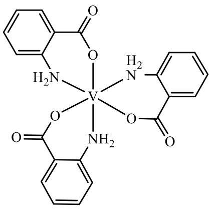

$V(anc)_3$

trianthrinilatovanadium(III)

11

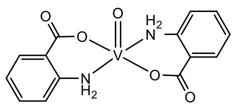

$VO(anc)_2$

dianthrinilatooxovanadium(IV)

12

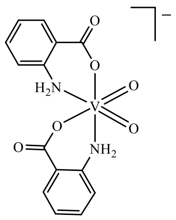

$[VO_2(anc)_2]^-$

dianthrinilatodioxovanadate(V)

13

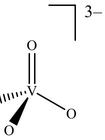

$[VO_4]^{3-}$

orthovanadate

14

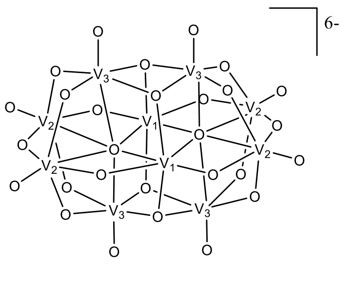

$[V_{10}O_{28}]^{6-}$

decavanadate

15

Figure S1. Graphical representation of complexes in Table 1.

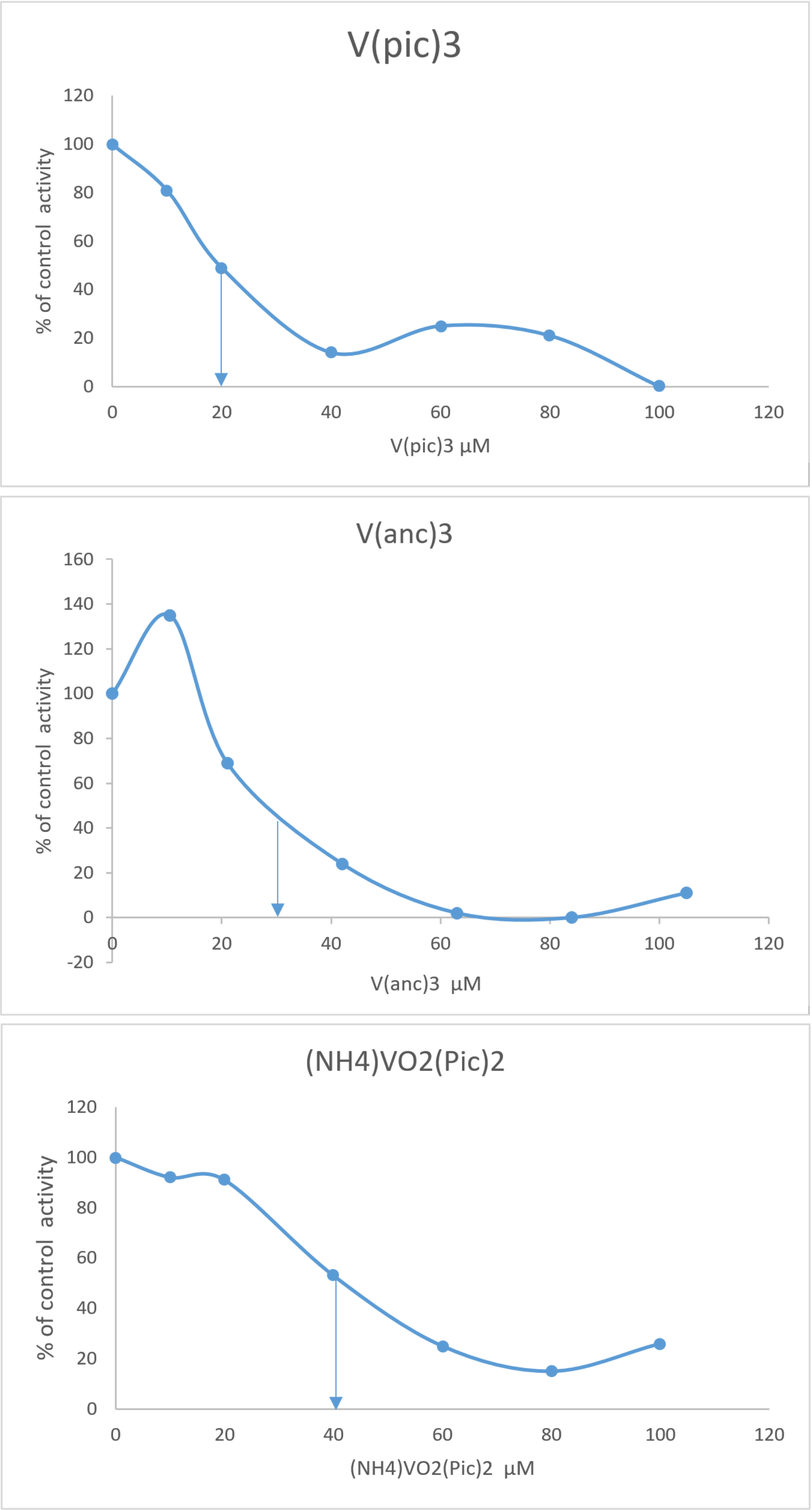

Figure S2. Plots for estimation of IC_{50} Values.
